# High-resolution melting curve FRET-qPCR rapidly distinguishes *Streptococcus equi* subsp. *equi* and *zooepidemicus*

**DOI:** 10.1128/spectrum.01529-25

**Published:** 2025-07-30

**Authors:** Nneka Vivian Iduu, Donna Raiford, Noah D. Cohen, Kerstin K. Landrock, Chengming Wang

**Affiliations:** 1 Molecular Diagnostic Laboratory, College of Veterinary Medicine, Auburn University 70721, Auburn, Alabama, USA; 2 Department of Pathobiology, College of Veterinary Medicine, Auburn University 70721, Auburn, Alabama, USA; 3 Bacteriology and Mycology Laboratory, College of Veterinary Medicine, Auburn University 70721, Auburn, Alabama, USA; 4 Department of Large Animal Clinical Sciences, College of Veterinary Medicine & Biomedical Sciences, Texas A&M University 67283, College Station, Texas, USA; South China Sea Institute of Oceanology, Chinese Academy of Sciences, Guangzhou, Guangdong, China

**Keywords:** *Streptococcus equi *subsp. *equi*, *Streptococcus equi* subsp. *zooepidemicus*, strangles, high-resolution melting curve analysis, FRET-qPCR, molecular diagnostics

## Abstract

We developed a fluorescence resonance energy transfer (FRET)-based real-time PCR (qPCR) assay coupled with high-resolution melting (HRM) analysis to differentiate *Streptococcus equi* subsp. *equi* (SEE) from *S. equi* subsp. *zooepidemicus* (SEZ), two closely related equine pathogens with distinct clinical implications. The assay targets a region of the 23S rRNA gene containing single-nucleotide polymorphisms that differentiate the subspecies. By combining subspecies-specific probes with melting temperature (*T*_m_) analysis, the assay accurately distinguished SEE, SEZ, and mixed infections without the need for post-PCR processing. The entire workflow—from DNA extraction to result interpretation—was completed in under 2 h. The assay demonstrated high sensitivity and specificity, detecting as few as one genome copy per reaction, and showed excellent reproducibility with 100% concordance to whole-genome sequencing (WGS) results. Notably, one nasal swab isolate biochemically identified as SEE (based on its fermentation profile) was reclassified as SEZ by the FRET-qPCR assay, a result confirmed by WGS. Among 127 equine clinical samples, SEE was detected in 18% (23/127) and SEZ in 82% (104/127). SEZ predominated in eye, sinus, transtracheal aspirate, uterine, and wound samples, whereas SEE was significantly associated with guttural pouch samples. This FRET-qPCR assay offers a rapid, accurate, and practical molecular diagnostic tool for distinguishing *S. equi* subspecies, supporting improved clinical management and outbreak control in equine populations.

**IMPORTANCE:**

Accurately identifying the cause of respiratory infections in horses is essential for proper treatment and preventing outbreaks. This study introduces a rapid and reliable test that distinguishes between *Streptococcus equi* subsp. *equi* (which causes the serious disease strangles) and its close relative *S. equi* subsp. *zooepidemicus* (which usually causes milder, less contagious infections). Traditional tests can be slow or give unclear results, especially for unusual strains. Our new one-step molecular test uses melting curve analysis to rapidly and accurately differentiate between these bacterial subspecies—even in mixed infections. The method, which matches the accuracy of whole-genome sequencing but is much faster and easier to use, can help veterinarians to make more accurate diagnoses and improve tracking and control of equine respiratory disease.

## OBSERVATION

*Streptococcus equi* subsp. *equi* (SEE) and *S. equi* subsp. *zooepidemicus* (SEZ) are closely related equine pathogens that differ significantly in their clinical impact. SEE is the causative agent of strangles, a highly contagious and sometimes fatal respiratory disease characterized by fever, nasal discharge, cough, and lymph node abscessation, with morbidity approaching 100% and mortality up to 10% (1–3). In contrast, SEZ is a commensal of the equine upper respiratory tract but can act as an opportunistic pathogen or primary pathogen, causing respiratory, reproductive, or systemic infections in horses and other species, including humans (4, 5). Both organisms are gram-positive, β-hemolytic streptococci classified under Lancefield group C and share approximately 98% genomic sequence identity (3, 6).

Respiratory tract infections in horses are a major veterinary concern worldwide, with significant consequences for animal welfare, athletic performance, and disease control efforts (4, 7, 8). Rapid and accurate identification of the causative agent is essential for guiding treatment, preventing transmission, and improving surveillance. However, differentiating SEE from SEZ remains challenging due to their phenotypic similarity and occasional mixed infections with *Streptococcus dysgalactiae* subsp. *equisimilis*, which further complicates diagnosis (9, 10).

Traditional diagnostic approaches rely on culturing β-hemolytic colonies followed by biochemical profiling, such as sugar fermentation tests. These methods are slow, often requiring 48 h or more and may yield ambiguous results due to inconsistent fermentation patterns and overlapping traits among strains, limiting the reliability of biochemical identification (10).

To address these limitations, molecular methods such as conventional PCR and real-time PCR (qPCR) assays have been developed, targeting genes including *seeI*, *seM*, and *sodA* (1, 11, 12). However, many of these assays rely on subspecies-specific primers or multiplex designs, which can reduce specificity, increase technical complexity, and limit their practicality in routine diagnostics (13, 14).

Fluorescence resonance energy transfer (FRET)-based qPCR combined with high-resolution melting (HRM) analysis offers a sensitive and specific strategy for detecting nucleotide polymorphisms within a closed-tube system (15). This approach enables real-time detection and post-amplification differentiation of closely related targets, reducing contamination risk and processing time.

In this study, we developed and validated a FRET-qPCR assay with HRM analysis to simultaneously detect and differentiate SEE and SEZ by targeting discriminatory single-nucleotide polymorphisms (SNPs) in 23S rRNA gene. Our goal was to establish a rapid, reliable, and cost-effective molecular diagnostic tool that overcomes the limitations of traditional assays and supports improved clinical diagnostics and epidemiological investigations in equine health.

A wide variety of equine clinical samples from sources including lymph node abscesses (9), eyes (14), gastrointestinal tract (1), guttural pouch (16), joints (4), lymph nodes (3), lungs (5), masses/tissues (4), muscle (3), nasal/nasopharyngeal region (17), sinuses (7), thoracic cavity (1), transtracheal aspirates (13), urethra and urine (3), uterus (18), veins (1), and wounds (9) were submitted to the Bacteriology and Mycology Diagnostic Laboratory at the College of Veterinary Medicine, Auburn University. In addition, five samples were submitted to the Diagnostic Laboratory at the Texas A&M College of Veterinary Medicine & Biomedical Sciences, College Station, Texas, USA, for the isolation of *Streptococcus* spp.

Bacterial cultures were grown on blood agar (BA), and isolates exhibiting smooth, translucent, shiny colonies with zones of β-hemolysis were selected for further analysis. Pure colonies were sub-cultured onto fresh BA plates and presumptively identified using Gram staining and the catalase test. Only gram-positive, catalase-negative isolates were retained for subsequent screening. These isolates were further characterized biochemically by inoculation into trehalose, salicin, sorbitol, and lactose fermentation broths for the differential identification of *Streptococcus* species and subspecies.

Based on the biochemical results, 23 isolates were identified as SEE, 104 as *Streptococcus equi* subsp. *zooepidemicus* (SEZ), and 14 non-target *Streptococcus* sp. (including *S. agalactiae* WT*, S. canis* WT*, S. pyogenes* ATCC 19615, *S. dysgalactiae* subsp. *equisimilis*). A total of 141 *Streptococcus* isolates and 6 non-*Streptococcus* isolates (including *Escherichia coli* 25922, *Staphylococcus aureus* 25923, and *Enterococcus faecalis* 29212) were blinded with respect to their species identification for further analysis.

Genomic DNA from the bacterial isolates was extracted using the IndiMag 2 automated magnetic bead-based nucleic acid isolation system (INDICAL Inc., Orlando, USA) with prefilled reagent cartridges, according to the manufacturer’s instructions. DNA was eluted in 100 µL of elution buffer and stored at −20 °C until use.

**Fig 1:**
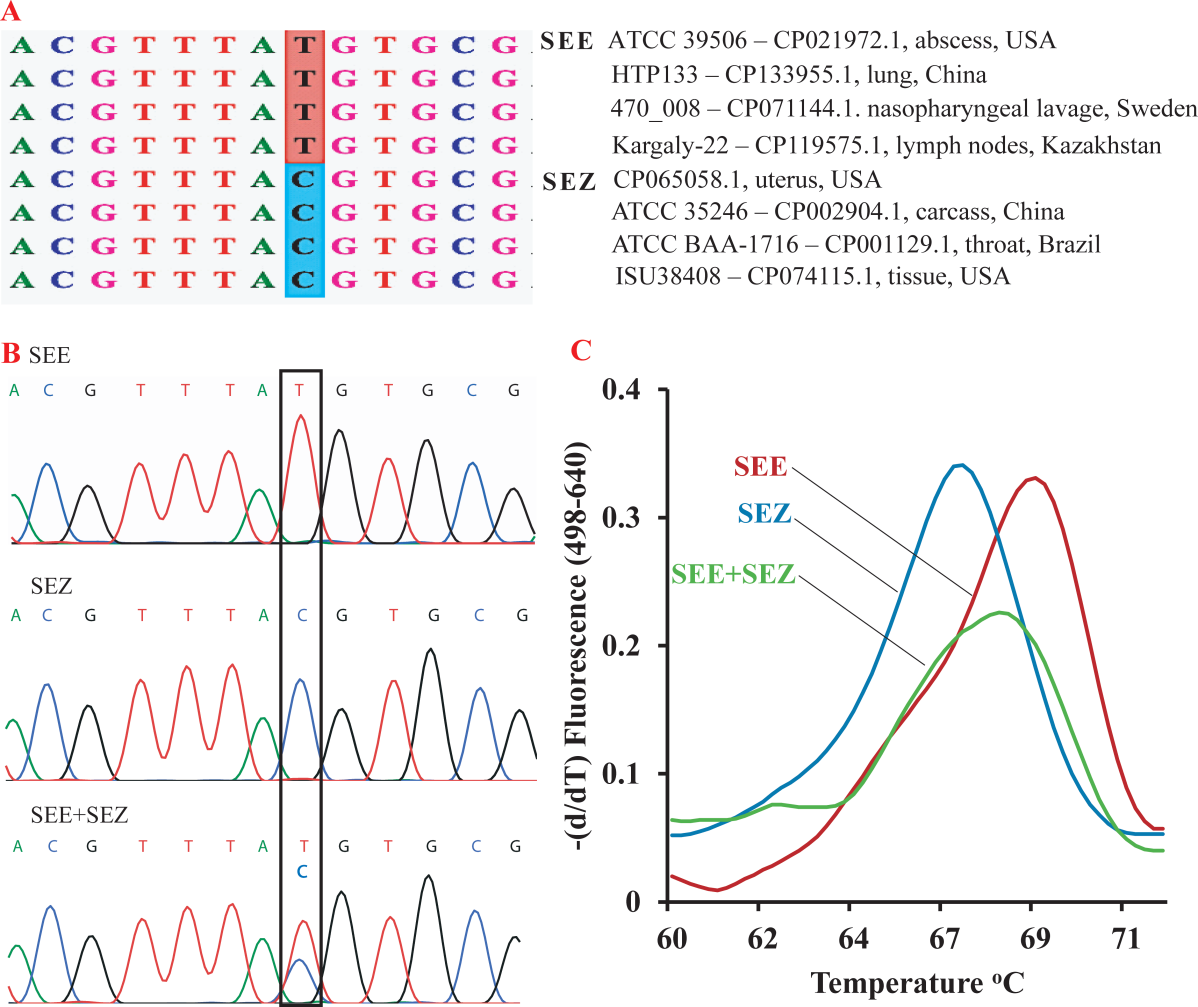
High-resolution melting FRET-qPCR analysis differentiates *S. equi* subsp. *equi* and (SEE) *S. equi* subsp. *zooepidemicus* (SEZ). (**A**) Multiple sequence alignment of representative SEE and SEZ genomes revealed a conserved single-nucleotide polymorphism (SNP) within the 23S rRNA gene, characterized by a thymine-to-cytosine substitution (T→C). gBlock DNA fragments containing the discriminatory SNPs were used as positive controls. (**B**) Sanger sequencing confirmed the target SNPs in SEE, SEZ, and mixed SEE + SEZ templates/bacterial DNAs. (**C**) High-resolution melting analysis clearly distinguished SEE (red, *T*_m_ ≈ 69°C), SEZ (blue, *T*_m_ ≈ 67°C), and mixed SEE + SEZ templates (green, *T*_m_ ≈ 68°C), based on characteristic melting profiles.

Representative genome sequences of SEE (CP021972.1, CP133955.1, CP071144.1, and CP119575.1) and SEZ (CP065058.1, CP002904.1, CP001129.1, and CP074115.1) were retrieved from NCBI GenBank and aligned using Vector NTI (Invitrogen, Carlsbad, CA, USA). A single-nucleotide polymorphism (SNP; T→C) in the 23S rRNA gene region was identified, differentiating SEE from SEZ (Fig. 1A). Conserved flanking regions around the SNP were analyzed using NCBI BLAST+v2.11.0, and primers were designed accordingly. A 6-carboxyfluorescein (6-FAM)-labeled hydrolysis probe was designed to target the discriminatory T nucleotide in SEE. *In silico* validation confirmed specificity, with no significant homology to other *Streptococcus* species, including *S. agalactiae*, *S. dysgalactiae*, and *S. pyogenes*.

Primers and probes (Integrated DNA Technologies, Coralville, IA, USA) targeting a 200 bp fragment included: forward primer: 5′-AGGGGAGTGAAAGAGAACCTGAAA-3′; reverse primer: 5′-ACATGGTTTSGGGTCTACGTCC-3′; anchor probe: 5′-GCGAGTTACGTTTATGTGCGAGGTTAAGT-6-FAM-3′; and reporter probe: 5′-Cy5-LCR640‐GAAGAGACGGAGCCGAAGGGAAAC-Phos-3′.

The anchor probe (FRET donor) was labeled with 6-FAM and excited at 488 nm; the reporter probe (FRET acceptor) was labeled with LC Red 640 and emitted at ~640 nm upon FRET excitation.

Thermal cycling differential *Streptococcus* FRET-PCR was performed in a LightCycler 480 II real-time PCR platform (Roche Diagnostics, Indianapolis, IN, USA). Each reaction was performed in a 20 µL final volume containing 10 µL of extracted DNA, and the thermal cycling consisted of 18 high-stringency step-down cycles followed by 30 relaxed-stringency fluorescence acquisition cycles as described by Gong et al. (15), with slight modification. The 18 high-stringency step-down thermal cycles were 6 × 10 s at 95°C, 12 s at 64°C, 8 s at 72°C; 9 × 10 s at 95°C, 12 s at 62°C, 8 s at 72°C; 3 × 10 s at 95°C, 12 s at 60°C, 8 s at 72°C. The relaxed-stringency fluorescence acquisition cycling consisted of 30 × 10 s at 95°C, followed by fluorescence acquisition of 12 s at 57°C and 10 s at 72°C.

The melting curve analysis for probe annealing to the PCR products was determined by monitoring the fluorescence from 45°C to 80°C, and fluorescence data were continuously collected using the F4/F1 emission ratio. The melting temperature (*T*_m_) was determined by plotting the negative derivative of fluorescence with respect to temperature (−d(F4/F1)/dT), generating a distinct melting peak corresponding to the dissociation of the FRET probe from its target sequence.

Analytical sensitivity was assessed using gBlock DNA fragments (Integrated DNA Technologies, Coralville, IA, USA) containing SEE- and SEZ-specific SNPs. Based on the molecular weight of each gBlock DNA fragment, 10-fold serial dilutions ranging from 10^4^ to 10^0^ copies per 10 µL reaction were prepared to determine the assay’s detection limit. Equal volumes of each dilution for SEE and SEZ gBlock, and for bacterial DNAs of SEE and SEZ (1:1 ratio) were also combined to evaluate the assay’s ability to detect both targets simultaneously. The specificity of the PCR was determined by testing the genomic DNA of the 14 non-target *Streptococcus* isolates to ensure the absence of cross-reactivity. In addition, the PCR products were sent to ELIM Biopharmaceuticals (Hayward, CA, USA) for bidirectional Sanger sequencing. The resulting sequence data were analyzed using ApE (v3.1.6).

Reproducibility was assessed using triplicate reactions of SEE and SEZ standards (10^4^ to 10^1^ copies/reaction). Intra-assay CV% was defined by the equation (standard deviation of triplicates/mean Ct) × 100%. Inter-assay CV% was defined as (standard deviation of mean Ct across runs/overall mean Ct) × 100%. The overall CV% was the average across all dilutions.

The DNA from all 147 bacterial isolates was analyzed using HRM FRET-qPCR to determine species identity (SEE, SEZ, or non-SEE/SEZ). Whole-genome sequencing (WGS) (Plasmidsaurus, Eugene, OR, USA) and DNA sequencing of the PCR products were performed on representative isolates for validation.

All statistical analyzes were conducted in RStudio (v4.4.3). Linear regression of the mean Ct values against log standard dilutions was used to evaluate the linearity of the qPCR. Site-specific distribution differences of SEE and SEZ in clinical samples were assessed using the exact binomial test. A *P* value ≤ 0.05 was considered statistically significant.

The developed FRET-qPCR demonstrated high analytical sensitivity, detecting as few as one gene copy per reaction. Linear regression across standard dilutions showed strong correlation (*R*^2^= 0.99) for both SEE and SEZ. Intra- and inter-assay reproducibility were high, with CV% values of 1.21% and 1.28% for SEE, and 1.35% and 1.41% for SEZ, respectively. Sanger sequencing confirmed the presence of the discriminatory SNP in both single (SEE or SEZ) and mixed PCR products/bacterial DNAs (SEE + SEZ) (Fig. 1B). This SNP produced distinct HRM profiles: SEZ exhibited a melting temperature (*T*_m_) of ~67 °C, SEE a *T*_m_ of ~69 °C, and mixed DNA a broader peak at ~68 °C (Fig. 1C). Melting peaks remained consistent across serial dilution. No amplification or melting peaks were observed for the negative control or 14 non-target *Streptococcus* isolates (*S. agalactiae* WT*, S. canis* WT*, S. pyogenes* ATCC 19615, and *S. dysgalactiae* subsp. *equisimilis*).

Of 147 bacterial isolates tested, the differential FRET-qPCR showed 100% concordance with WGS and DNA sequencing, and 99% agreement with biochemical identification (146/147). SEE was detected in 23 out of 127 *Streptococcus equi* isolates (18%), and SEZ in 104 isolates (82%). All six non-*Streptococcus* isolates (*Escherichia coli* 25922, *Staphylococcus aureus* 25923, and *Enterococcus faecalis* 29212) were correctly identified as negative by differential PCR. One nasal swab isolate with an ambiguous fermentation profile (lactose and salicin-positive, trehalose- and sorbitol-negative) was identified biochemically as SEE but was identified by FRET-qPCR as SEZ. WGS and PCR product sequencing confirmed SEZ, validating the FRET-qPCR result.

**Fig 2:**
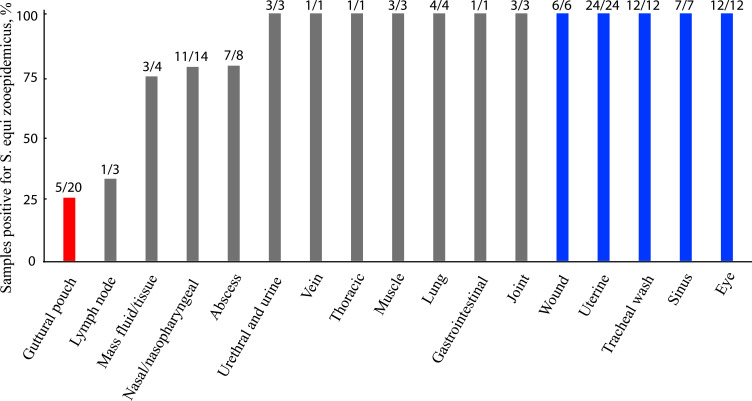
Prevalence of *S. equi* subsp. *equi* (SEE) and *S. equi* subsp. *zooepidemicus* (SEZ) in equine clinical samples. The proportions of SEZ were calculated relative to the total number of SEE and SEZ isolates recovered from each anatomical site, as indicated on top of each bar. Binomial tests were used to compare the detection rates of SEE and SEZ across the various clinical sample sources, with statistical significance set at *P* ≤ 0.05. SEZ (blue bars) was significantly more prevalent in samples from the eye (95% CI: 0–26.5%, *P* = 0.00049), sinus (95% CI: 0–41%, *P* = 0.0156), transtracheal aspirates (95% CI: 0–26.5%, *P* = 0.00049), uterus (95% CI: 0–14.2%, *P* = 1.19 × 10⁻⁷), and wounds (95% CI: 0–45.9%, *P* = 0.0313). In contrast, SEE (red bar) was significantly more common in guttural pouch samples (95% CI: 50.9–91.3%, *P* = 0.0414). No significant differences (gray bars) were observed in other clinical sample types (*P* > 0.05).

Distribution analysis revealed SEE was significantly more prevalent in guttural pouch samples (15/20; 95% CI: 50.9–91.3%, *P* = 0.0414). In contrast, SEZ was significantly more common in samples from the eye (12/12; 95% CI: 0–26.5%, *P* = 0.00049), sinus (7/7; 95% CI: 0–41%, *P* = 0.0156), transtracheal aspirates (12/12; 95% CI: 0–26.5%, *P* = 0.00049), uterus (24/24; 95% CI: 0–14.2%, *P* = 1.19 × 10⁻⁷), and wounds (6/6; 95% CI: 0–45.9%, *P* = 0.0313), with no detection of SEE in these sample types (Fig. 2).

SEE, a host-adapted clone that evolved from the more genetically diverse SEZ, is responsible for strangles—an endemic and economically impactful disease in equine populations (17, 19). Accurate and timely differentiation between SEE and SEZ remains diagnostically challenging, particularly when relying on phenotypic characteristics such as sugar fermentation profiles. While SEE typically does not ferment lactose, sorbitol, or trehalose, SEZ can ferment lactose and sorbitol but not trehalose (9). However, genomic plasticity in SEZ, likely driven by horizontal gene transfer, can lead to atypical biochemical phenotypes (20, 21), such as the sorbitol-negative SEZ isolate observed in this study. Despite this phenotypic variation, the conserved genomic targets used in our assay—unaffected by such variability—allowed accurate genotypic identification, a finding further confirmed by WGS.

The ability of this assay to resolve such discrepancies highlights a significant advantage over conventional culture-based techniques, which may misclassify atypical strains or fail to detect co-infections. Although some inconsistencies in previous reports were attributed to mixed infections (13), this explanation was excluded here due to the use of pure isolate cultures and WGS verification.

This study presents the development and technical validation of a rapid, accurate, and convenient FRET-qPCR assay with HRM analysis for the simultaneous detection and differentiation of SEE and SEZ. By targeting discriminatory SNPs within the 23S rRNA gene, the assay achieves clear subspecies identification based on distinct melting curve profiles within a single closed-tube reaction. This approach minimizes contamination risk, simplifies the diagnostic workflow, and offers results in under 2 h, which is significantly faster than conventional biochemical methods that require up to 48 h (12, 22).

Compared to previously established PCR assays, which often rely on subspecies-specific primers or multiplex designs, our assay provides dual detection without the need for multiple reactions or post-PCR processing. Earlier methods also face limitations, including potential gene truncations, cross-reactivity with non-target *Streptococci*, and an inability to detect concurrent infections with SEE and SEZ (11, 23). In contrast, the FRET-based HRM technique employed here enables sensitive detection of SNPs and robust differentiation between subspecies, even in mixed infections, using a single primer-probe set.

The low intra- and inter-assay coefficients of variation—both well below 5%—highlight the assay’s high reproducibility, which is critical for routine diagnostics and reliable quantification (16). This reproducibility further strengthens its potential utility in veterinary laboratories, particularly in settings requiring high-throughput testing or epidemiological screening.

Moreover, our findings reflect differing patterns in anatomical distribution between SEE and SEZ. SEE isolates were significantly more likely to be recovered from guttural pouch samples, consistent with prior reports of their ability to persist in this site after clinical recovery, often within chondroids that act as reservoirs for bacterial shedding and transmission (1, 24).

SEZ is a known zoonotic pathogen capable of causing severe infections in humans, including meningitis, septicemia, and respiratory illness, particularly in individuals with close contact with animals. SEZ was identified across a broader array of clinical specimens, including ocular, sinus, uterine, and wound samples, supporting its role as a versatile opportunist capable of colonizing multiple mucosal surfaces and invading tissues under conditions of host compromise (6, 18). In this study, SEZ was detected in 100% of the ocular samples tested, highlighting its high prevalence and potential role in ocular infections. This finding underscores the importance of monitoring SEZ in both veterinary and public health contexts, given its zoonotic risk. Its recovery from ocular samples, in particular, raises concern, as SEZ has been implicated not only in equine eye infections but also in rare, severe ocular infections in humans such as endophthalmitis, with zoonotic transmission suspected in some cases (25–27). Its recovery from ocular samples, in particular, raises concern, as SEZ has been implicated not only in equine eye infections but also in rare, severe ocular infections in humans such as endophthalmitis, with zoonotic transmission suspected in some cases (25–27). This emphasizes the importance of monitoring SEZ in both veterinary and public health settings.

While this study demonstrates the FRET-qPCR assay’s robustness using pure cultures and controlled co-infection models, further diagnostic validation is needed in clinical settings where naturally occurring mixed infections may exhibit more complex dynamics. Additionally, the limited number of samples from certain anatomical sites restricts definitive conclusions regarding the full distribution patterns of these subspecies and warrants larger, geographically diverse studies.

The FRET-qPCR assay with HRM described here represents a significant advancement in equine infectious disease diagnostics. Its rapid turnaround, high sensitivity, and ability to distinguish between SEE and SEZ in a single reaction make it a valuable tool for veterinary laboratories and potential point-of-care settings. Early and accurate identification can inform targeted biosecurity measures, helping contain strangles outbreaks while avoiding unnecessary interventions for generally less contagious SEZ infections.

In the future, this platform could be adapted to study *S. equi* subspecies infections in other animal hosts, especially given SEZ’s zoonotic potential. Additional research may also explore the assay’s use in direct clinical specimen testing, bypassing culture steps to further streamline diagnostics. Finally, integrating SNP-based diagnostic tools like this with broader genomic surveillance could enhance tracking of outbreak sources and the emergence of novel virulent strains, thus improving disease management at both local and global levels.

## References

[1] Boyle AG, Rankin SC, Duffee L, Boston RC, Wheeler-Aceto H. 2016. Streptococcus equi detection polymerase chain reaction assay for equine nasopharyngeal and guttural pouch wash samples. J Vet Intern Med 30:276–281. doi:10.1111/jvim.13808 26678318 PMC4913660

[2] Holden MTG, Heather Z, Paillot R, Steward KF, Webb K, Ainslie F, Jourdan T, Bason NC, Holroyd NE, Mungall K, et al.. 2009. Genomic evidence for the evolution of Streptococcus equi: host restriction, increased virulence, and genetic exchange with human pathogens. PLoS Pathog 5:e1000346. doi:10.1371/journal.ppat.1000346 19325880 PMC2654543

[3] Rotinsulu DA, Ewers C, Kerner K, Amrozi A, Soejoedono RD, Semmler T, Bauerfeind R. 2023. Molecular features and antimicrobial susceptibilities of Streptococcus equi ssp. equi isolates from strangles cases in Indonesia. Vet Sci 10:49. doi:10.3390/vetsci10010049 36669050 PMC9867300

[4] Lindahl SB, Aspán A, Båverud V, Paillot R, Pringle J, Rash NL, Söderlund R, Waller AS. 2013. Outbreak of upper respiratory disease in horses caused by Streptococcus equi subsp. zooepidemicus ST-24. Vet Microbiol 166:281–285. doi:10.1016/j.vetmic.2013.05.006 23773239

[5] Mitchell CM, Johnson LK, Crim MJ, Wiedmeyer CE, Pugazhenthi U, Tousey S, Tollin DJ, Habenicht LM, Fink MK, Fong DL, Leszczynski JK, Manuel CA. 2020. Diagnosis, Surveillance and Management of Streptococcus equi subspecies zooepidemicus Infections in Chinchillas (Chinchilla lanigera). Comp Med 70:370–375. doi: 10.30802/AALAS-CM-20-000012 32731906 10.30802/AALAS-CM-20-000012 PMC7446643

[6] Timoney JF. 2004. The pathogenic equine streptococci. Vet Res 35:397–409. doi:10.1051/vetres:2004025 15236673

[7] Javed R, Taku AK, Gangil R, Sharma RK. 2016. Molecular characterization of virulence genes of Streptococcus equi subsp. equi and Streptococcus equi subsp. zooepidemicus in equines. Vet World 9:875–881. doi:10.14202/vetworld.2016.875-881 27651677 PMC5021838

[8] Mańkowska A, Witkowska D. 2024. The most common environmental risk factors for equine asthma-a narrative review. Animals (Basel) 14:2062. doi:10.3390/ani14142062 39061524 PMC11273653

[9] Chhabra D, Nagra J, Manuja A, Singha HS, Vaid RK, Goutam U, Kumar B. 2025. Phenotypic, biochemical and molecular characterization of Streptococcus equi isolates in Northern India. Indian J Microbiol 65:1292–1298. doi:10.1007/s12088-024-01420-5 40655359 PMC12246333

[10] Webb K, Barker C, Harrison T, Heather Z, Steward KF, Robinson C, Newton JR, Waller AS. 2013. Detection of Streptococcus equi subspecies equi using a triplex qPCR assay. The Veterinary Journal 195:300–304. doi:10.1016/j.tvjl.2012.07.007 22884566 PMC3611602

[11] Cordoni G, Williams A, Durham A, Florio D, Zanoni RG, La Ragione RM. 2015. Rapid diagnosis of strangles (Streptococcus equi subspecies equi) using PCR. Res Vet Sci 102:162–166. doi:10.1016/j.rvsc.2015.08.008 26412537

[12] Noll LW, Stoy CPA, Wang Y, Porter EG, Lu N, Liu X, Burklund A, Peddireddi L, Hanzlicek G, Henningson J, Chengappa MM, Bai J. 2020. Development of a nested PCR assay for detection of Streptococcus equi subspecies equi in clinical equine specimens and comparison with a qPCR assay. J Microbiol Methods 172:105887. doi:10.1016/j.mimet.2020.105887 32165161

[13] Morris ERA, Schroeder ME, Ferro PJ, Waller AS, McGlennon AA, Bustos CP, Gressler LT, Wu J, Lawhon SD, Boyle AG, Lingsweiler S, Paul N, Dimitrov K, Swinford AK, Bordin AI, Cohen ND. 2023. Development of a novel real-time PCR multiplex assay for detection of Streptococcus equi subspecies equi and Streptococcus equi subspecies zooepidemicus. Vet Microbiol 284:109797. doi:10.1016/j.vetmic.2023.109797 37290208

[14] Pusterla N, Leutenegger CM, Barnum SM, Byrne BA. 2018. Use of quantitative real-time PCR to determine viability of Streptococcus equi subspecies equi in respiratory secretions from horses with strangles. Equine Vet J 50:697–700. doi:10.1111/evj.12809 29341315

[15] Gong J, Iduu NV, Zhang D, Chenoweth K, Wei L, Yang Y, Dou X, Wang C. 2024. Dual-emission fluorescence resonance energy transfer (FRET) PCR discriminates Salmonella Pullorum and Gallinarum. Microorganisms 12:1815. doi:10.3390/microorganisms12091815 39338489 PMC11433795

[16] Chengula AA, Mugimba KK, Tal S, Levi RT, Dubey S, Mutoloki S, Dishon A, David L, Evensen Ø, Munang’andu HM. 2022. Efficiency, sensitivity and specificity of a quantitative real-time PCR assay for Tilapia Lake virus (TiLV). J Virol Methods 307:114567. doi:10.1016/j.jviromet.2022.114567 35709972

[17] Wilson HJ, Dong J, van Tonder AJ, Ruis C, Lefrancq N, McGlennon A, Bustos C, Frosth S, Léon A, Blanchard AM, Holden M, Waller AS, Parkhill J. 2025. Progressive evolution of Streptococcus equi from Streptococcus equi subsp. zooepidemicus and adaption to equine hosts. Microb Genom 11. doi:10.1099/mgen.0.001366 PMC12453393 40152912

[18] Clark C, Greenwood S, Boison JO, Chirino-Trejo M, Dowling PM. 2008. Bacterial isolates from equine infections in western Canada (1998-2003). Can Vet J Rev Veterinaire Can 49:153–160. PMC2216435 18309745

[19] Preziuso S, Moriconi M, Cuteri V. 2019. Genetic diversity of Streptococcus equi subsp. zooepidemicus isolated from horses. Comp Immunol Microbiol Infect Dis 65:7–13. doi:10.1016/j.cimid.2019.03.012 31300129

[20] Azpiroz MF, Burger N, Mazza M, Rodríguez G, Camou T, García Gabarrot G. 2023. Characterization of Streptococcus equi subsp. zooepidemicus isolates containing lnuB gene responsible for the L phenotype. PLoS One 18:e0284869. doi:10.1371/journal.pone.0284869 37115801 PMC10146458

[21] Su Y, Zhang Z, Wang L, Zhang B, Su L. 2024. Whole-genome sequencing and phenotypic analysis of Streptococcus equi subsp. zooepidemicus sequence type 147 isolated from China. Microorganisms 12:824. doi:10.3390/microorganisms12040824 38674768 PMC11051846

[22] Boyle AG, Timoney JF, Newton JR, Hines MT, Waller AS, Buchanan BR. 2018. Streptococcus equi infections in horses: guidelines for treatment, control, and prevention of strangles—revised consensus statement . Veterinary Internal Medicne 32:633–647. doi:10.1111/jvim.15043 PMC5867011 29424487

[23] Alber J, El‐Sayed A, Lämmler C, Hassan AA, Weiss R, Zschöck M. 2004. Multiplex polymerase chain reaction for identification and differentiation of Streptococcus equi subsp. zooepidemicus and Streptococcus equi subsp equi. J Vet Med Ser 51:455–458. doi:10.1111/j.1439-0450.2004.00799.x 15606870

[24] Waller AS. 2014. New perspectives for the diagnosis, control, treatment, and prevention of strangles in horses. Vet Clin North Am Equine Pract 30:591–607. doi:10.1016/j.cveq.2014.08.007 25300634

[25] Deniaud M, Tee E. 2023. Susceptibility pattern of bacterial isolates in equine ulcerative keratitis: implications for empirical treatment at a university teaching hospital in Sydney. Aust Vet J 101:115–120. doi:10.1111/avj.13221 36433648

[26] Madžar D, Hagge M, Möller S, Regensburger M, Lee D-H, Schwab S, Jantsch J. 2015. Endogenous endophthalmitis complicating Streptococcus equi subspecies zooepidemicus meningitis: a case report. BMC Res Notes 8:184. doi:10.1186/s13104-015-1133-9 25940309 PMC4423494

[27] Morris RE, Doherty S, Oltmanns MH, Sapp MR, Wells K, Patel HR. 2024. Horse to human: Streptococcus equi septicemia presenting as endogenous endophthalmitis. Am J Ophthalmol Case Rep 33:101974. doi:10.1016/j.ajoc.2023.101974 38292882 PMC10825363

